# Development of an attenuated potato virus Y mutant carrying multiple mutations in helper-component protease for cross-protection

**DOI:** 10.1016/j.virusres.2024.199369

**Published:** 2024-04-15

**Authors:** Xiao-Jie Xu, Xu-Jie Sun, Chun-Ju Liu, Xiu-Zhai Chen, Qing Zhu, Yan-Ping Tian, Xiang-Dong Li

**Affiliations:** a The Engineering Research Institute of Agriculture and Forestry, Ludong University, Yantai, Shandong 264025, China; b Laboratory of Plant Virology, Department of Plant Pathology, College of Plant Protection, Shandong Agricultural University, Tai'an, Shandong 271018, China; c Weifang Tobacco Corporation, Weifang, Shandong 261031, China; d Linyi Tobacco Corporation, Linyi, Shandong 276000, China

**Keywords:** Cross-protection, Helper component-protease, Potato virus Y, Site-directed mutagenesis, Stability

## Abstract

Tobacco (*Nicotiana tabacum*) is one of the major cash crops in China. Potato virus Y (PVY), a representative member of the genus *Potyvirus*, greatly reduces the quality and yield of tobacco leaves by inducing veinal necrosis. Mild strain-mediated cross-protection is an attractive method of controlling diseases caused by PVY. Currently, there is a lack of effective and stable attenuated PVY mutants. Potyviral helper component-protease (HC-Pro) is a likely target for the development of mild strains. Our previous studies showed that the residues lysine at positions 124 and 182 (K^124^ and K^182^) in HC-Pro were involved in PVY virulence, and the conserved KITC motif in HC-Pro was involved in aphid transmission. In this study, to improve the stability of PVY mild strains, K at position 50 (K^50^) in KITC motif, K^124^, and K^182^ were separately substituted with glutamic acid (E), leucine (L), and arginine (R), resulting in a triple-mutant PVY-HC_ELR_. The mutant PVY-HC_ELR_ had attenuated virulence and did not induce leaf veinal necrosis symptoms in tobacco plants and could not be transmitted by *Myzus persicae*. Furthermore, PVY-HC_ELR_ mutant was genetically stable after six serial passages, and only caused mild mosaic symptoms in tobacco plants even at 90 days post inoculation. The tobacco plants cross-protected by PVY-HC_ELR_ mutant showed high resistance to the wild-type PVY. This study showed that PVY-HC_ELR_ mutant was a promising mild mutant for cross-protection to control PVY.

## Highlights

• A non-aphid-transmissible attenuated PVY triple-mutant PVY-HC_ELR_ was developed.
• PVY-HC_ELR_ mutant was stable in tobacco plants.
• PVY-HC_ELR_ mutant protected tobacco plants from the severe PVY infection.
• Provided the material basis for the management of PVY through cross-protection.

## 1 Introduction

China is the largest producer of tobacco (*Nicotiana tabacum*) leaves in the world (Ahmed et al., 2022; Fang et al., 2019). Tobacco is an economic crop susceptible to many plant viruses including tobacco mosaic virus (TMV), tobacco etch virus (TEV), tobacco vein banding mosaic virus (TVBMV), and potato virus Y (PVY) (Eckel and Lampert, 1993; Lewis, 2007; Scholthof, 2004; Tian et al., 2007; Zhao et al., 2023). PVY has evolved into different groups, including two main N and O groups. Group O induces mosaic and mottle symptoms, while group N causes systemic veinal necrosis symptoms on tobacco leaves (Karasev and Gray, 2013; McDonald and Singh, 1996; Nie et al., 2012; Tian and Valkonen, 2013), resulting in significant yield losses to tobacco production (Faurez et al., 2012; Hu et al., 2009; Tsedaley, 2015). A growing number of PVY recombinant strains emerged in China, such as PVY^SYR-I^, PVY^SYR-II^, PVY^SYR-III^, PVY^N-Wi^, PVY^NTN^, and PVY^N:O^ (Bai et al., 2019; Ding et al., 2022; Gao et al., 2024).

Cross-protection is a promising measure to control plant viral diseases and has been used for the management of more than 35 plant viruses (Pechinger et al., 2019; Zhang and Qu, 2016; Ziebell and Carr, 2010). There are two major models of the mechanisms underlying cross-protection: superinfection exclusion and RNA silencing (Guo et al., 2016; Zhang et al., 2018). The stability of mild strain is an essential requirement for the successful use of cross-protection in the field (Ziebell and MacDiarmid, 2017). However, the polymerases of RNA viruses lack proofreading activity, leading to constant mutations in their genomes (Domingo et al., 2021; Roossinck, 1997). Consequently, some mild strains may evolve into virulent strains during their replication (Hasiów-Jaroszewska et al., 2011; Loebenstein and Carr, 2006). The substitution of valine at position 192 (V^192^) in helper component-protease (HC-Pro) with alanine (A) reduced the virulence of TEV. A compensatory mutation of tyrosine at position 642 (Y^642^) to C restored TEV virulence (Torres-Barceló et al., 2009). Virus mild mutants carrying multiple mutations are less likely to become virulent compared to those carrying a single mutation (Goławski et al., 2022; Maassab and DeBorde, 1985; Tosh et al., 2008), and have been used for cross-protection against zucchini yellow mosaic virus (ZYMV), sugarcane mosaic virus (SCMV), potato virus X (PVX), and papaya leaf distortion mosaic virus (PLDMV) (Cong et al., 2019; Lin et al., 2007; Tuo et al., 2020; Xu et al., 2021). However, PVY mild strains containing multiple attenuating mutations are still scarce up to now.

PVY belongs to the genus *Potyvirus* (family *Potyviridae*), and is transmitted by at least 25 aphid species in a non-persistent manner, of which *Myzus persicae* is considered the most efficient vector (Boquel et al., 2011; Mello et al., 2011). The single-stranded RNA genome of PVY encodes at least 11 proteins (Chung et al., 2008; Olspert et al., 2015). HC-Pro is a major factor of PVY determining the symptom of tobacco veinal necrosis. The residues lysine at position 400 (K^400^) and glutamic acid at position 419 (E^419^) of PVY^N-605^ isolate HC-Pro are reported to be involved in causing vein necrosis symptoms in tobacco plants (Faurez et al., 2012). The substitution of aspartic acid at position 205 (D^205^) in HC-Pro with glycine (G) renderes the PVY^NTN^ isolates incapable of inducing the tobacco venous necrosis phenotype (Hu et al., 2009). The amino acid residues K at position 391 (K^319^) and E at position 410 (E^410^) in HC-Pro are also veinal necrosis determinants for PVY^SYR-I^ isolate (Cheng et al., 2020). Furthermore, potyviral HC-Pro is also involved in aphid transmission, RNA silencing suppression, and virulence. The mutation of K in the conserved KITC motif of HC-Pro to E results in a decrease in aphid transmission of plum pox virus, tobacco vein mottling virus, and PVY (Atreya and Pirone, 1993; Blanc et al., 1998; Goytia et al., 2006). The substitution of conserved residues cysteine at positions 57 and 60 (C^57^ and C^60^) in the zinc finger-like motif of HC-Pro with A reduces its RNA silencing suppression activity and the virulence of sugarcane mosaic virus (SCMV) (Xu et al., 2021). The substitution of conserved residue R in HC-Pro FRNK motif with isoleucine (I) reduces the virulence of SCMV, TVBMV, and ZYMV (Gao et al., 2012; Shiboleth et al., 2007; Xu et al., 2020). Therefore, HC-Pro is an important target for screening a promising mild mutant for cross-protection of potyviruses.

In our previous study, we have shown that the residue K at position 124 (K^124^) is involved in the RNA silencing suppression activity of HC-Pro and PVY virulence, and the residue K at position 182 (K^182^) in HC-Pro FRNK motif of PVY is a critical amino acid responsible for tobacco veinal necrosis (Tuo, 2016; Zhang et al., 2021). Based on these studies, we developed a promising attenuated PVY mutant with triple mutations in HC-Pro, and evaluated its aphid transmission rate, stability, and cross-protective potential in tobacco plants. This study provides an environmentally safe measure to control PVY in tobacco plants.

## 2 Materials and methods

### 2.1 Plant growth

Plants of tobacco (*N. tabacum* cv. Xanthi) were grown in an environment-controlled chamber at 24°C, 125 μmol·m^−2^·s^−^^1^ light intensity, 16 hours (h) light: 8 h dark.

### 2.2 Plasmid construction and virus inoculation

The infectious clone of the wild-type PVY (pPVY) based on PVY isolate Guizhou (GenBank accession: MN381731) and the GFP-expressing infectious clone of PVY (pPVY-GFP) were constructed in the Laboratory of Plant Virology, Shandong Agricultural University (Cheng et al., 2020). PVY mutants were constructed by the site-directed mutagenesis method (Liu and Naismith, 2008) using the primers listed in Supplemental Table 1.

The plasmids of PVY and its mutants were separately introduced into *Agrobacterium tumefaciens* cells GV3101 by heat-shock transformation (Hofgen and Willmitzer, 1988). The transformed *Agrobacterium* cells were prepared and infiltrated to the lower two fully expanded leaves of tobacco plants essentially as described by Cheng *et al*. (2020). Crude extracts from tobacco leaves infected with PVY-GFP were ground in 0.02 M phosphate-buffered saline (pH 7.2) at a 1:5 (w/v) ratio. The crude extracts were rub-inoculated to the first fully expanded leaves of tobacco plants. The experiments were repeated three times.

### 2.3 RNA extraction and semi-quantitative reverse transcription polymerase chain reaction (SqRT-PCR)

The total RNA was extracted from tobacco leaves using TRIzol reagent (TransGen Biotech, Beijing, China). 500 ng total RNA was used for the first-strand cDNA synthesis by HiScript II Q RT select SuperMix (+gDNA wiper; Vazyme, Nanjing, China). 25 ng cDNA was used for sqRT-PCR analysis in a 20 μL reaction volume. The PCR procedure was carried out as follows: denaturation at 94°C for 5 minutes (min), followed by 22 cycles of 94°C/30 seconds (s), 60°C/30 s, 72°C/45 s, with a final extension of 72°C/5 min. The housekeeping genes *18S rRNA* (GenBank accession: HQ384692) and *EF1α* (GenBank accession: XM009784954) in tobacco plants were used as the internal controls (Ellis et al., 2020; Michel et al., 2019). The experiments were repeated three times.

### 2.4 Western blotting

Total proteins were extracted from tobacco leaves and separated on 12 % SDS–PAGE gels, then transferred onto nitrocellulose membranes (Pall Gelman, New York, USA) (Sun and Suzuki, 2008). Blotted membranes were probed with the rabbit polyclonal antiserums against PVY CP or GFP prepared in the Laboratory of Plant Virology, Shandong Agricultural University, and then with goat anti-rabbit IgG conjugated with horseradish peroxidase (Sigma-Aldrich, St. Louis, MO, USA). Detection was carried out with the SuperSignal™ West Dura Extended Duration Substrate solution (Thermo Fisher Scientific). The intensity of each band was evaluated using the ImageJ software (Wyrsch et al., 2015). The experiments were repeated three times.

### 2.5 Leaf veinal necrosis detection

For veinal necrosis analyses, the upper non-inoculated leaves of tobacco plants were stained with lactophenol-trypan blue as described previously (García-Marcos et al., 2013).

### 2.6 Aphid transmission assays

The aphid transmission assays were performed as shown in Fig. S1. Green peach aphids (*M. persicae*) were starved for 3 h, then fed with tobacco leaves infected with PVY-GFP or its mutant for 5 min. Thereafter, groups of five aphids were transferred to healthy tobacco plants to feed for 12 h, and then the aphids were killed by spraying the plants with imidacloprid (0.09 g/L). The *M. persicae* transmission rates of PVY-GFP and its mutant were detected by SqRT-PCR using gene-specific primers for PVY CP.

### 2.7 Stability test

Tobacco plants were first infiltrated with *Agrobacterium* cultures carrying the plasmid of PVY mutant, and then crude extracts from the systemic leaves infected with PVY mutant were mechanically inoculated onto healthy tobacco plants. Six serial passages were performed at a 20-day interval. After three and six serial passages, the HC-Pro coding sequences of PVY mutant progeny in tobacco plants were sequenced.

### 2.8 Cross-protection assay

Tobacco plants were pre-inoculated with the *Agrobacterium* cultures harboring the plasmid of the PVY mutant. At 5 and 10 days after the protective inoculation with PVY mutant, the latest fully expanded tobacco leaves were challenged with saps from tobacco leaves infected with PVY-GFP. Tobacco plants infiltrated with *Agrobacterium* cells carrying the empty vector pCB301-Rz were used as non-protected controls. At 15 days after challenge inoculation, the cross-protection efficacy was evaluated by measuring PVY-GFP accumulation through western blotting using a primary antibody against GFP, and SqRT-PCR using gene-specific primers for *GFP*. The experiments were repeated three times.

## 3 Results

### 3.1 PVY triple-mutant results in attenuated symptoms in tobacco plants

**Fig. 1:**
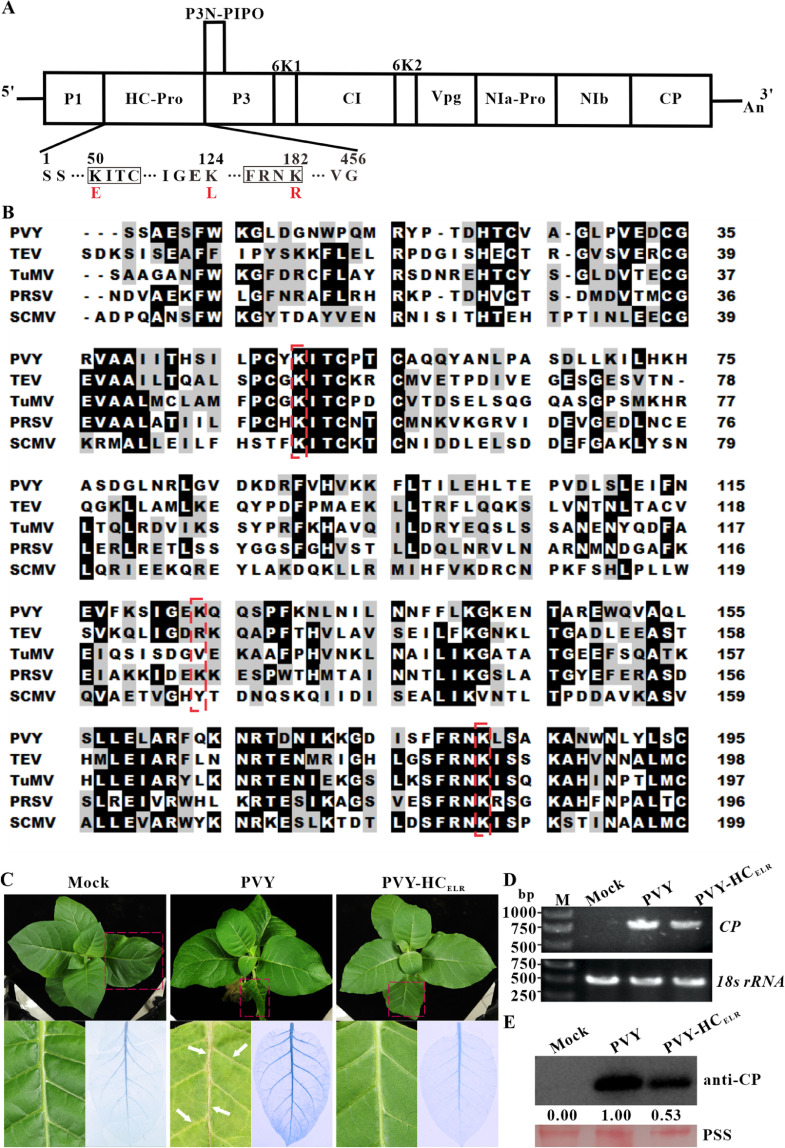
**Symptoms and accumulation of attenuated variant of potato virus Y (PVY) in tobacco plants.** (A) Schematic representation of PVY genome, showing all the mutations in helper component protease (HC-Pro). The conserved KITC and FRNK motifs in PVY HC-Pro were marked with black boxes. The red letters were the substituted residues in PVY HC-Pro. (B) Alignment of partial amino acid sequences of five potyviral HC-Pros using the BioEdit program version 7.2.5. The amino acid residues lysine at position 50 (K^50^) in the conserved KITC motif, K at position 124 (K^124^), and K at position 182 (K^182^) in the conserved FRNK motif of HC-Pro were marked with red-dotted boxes. The analysis was performed with HC-Pro sequences of PVY isolate Guizhou (GenBank accession: MN381731), tobacco etch virus (TEV, GenBank accession: DQ986288), turnip mosaic virus (TuMV, GenBank accession: AB093596), papaya ringspot virus (PRSV, GenBank accession: MF085000), and sugarcane mosaic virus (SCMV, GenBank accession: AY042184). (C) Symptoms of tobacco (*Nicotiana tabacum* cv. Xanthi) plants infected with the wild-type PVY and its mutant. Mock, tobacco plants inoculated with the empty vector pCB301-Rz. PVY, tobacco plants infected with the wild-type PVY. The residues of K^50^, K^124^, and K^182^ were separately substituted with glutamic acid (E), leucine (L), and arginine (R) in HC-Pro of PVY-HC_ELR_ mutant. Leaf veinal necrosis of tobacco plants under daylight and staining with trypan blue. (D) Semi-quantitative reverse-transcriptase polymerase chain reaction (SqRT-PCR) analysis of the RNA accumulation levels of PVY CP in tobacco upper leaves infected with PVY and PVY-HC_ELR_ mutant at 15 days post agroinfiltration (dpai). 18S ribosomal RNA (18S rRNA) was used as an internal control. (E) Western blotting analysis of the wild-type and mutant PVY CP accumulation levels in tobacco upper leaves at 15 dpai. The sample loadings were shown with the ponceau S staining (PSS). Band intensities were measured using the ImageJ software. Numbers indicated PVY CP accumulation levels normalized to PSS staining. The experiments were repeated three times.

To obtain attenuated PVY mutant, we introduced three mutations in PVY HC-Pro, as shown in Fig. 1A, by using site-directed mutagenesis primers listed in Supplementary Table 1. The resulting PVY triple mutant plasmid was named as pPVY-HC_ELR_. The mutant plasmid was transformed into *Agrobacterium* tumefaciens cells and infiltrated to the fully expanded leaves of tobacco plants. The HC-Pro of PVY-HC_ELR_ progeny had three mutations: the residue K at position 50 (K^50^) in the conserved KITC motif was substituted with E, K at position 124 (K^124^) was substituted with leucine (L), and K at position 182 (K^182^) in the conserved FRNK motif was substituted with R (Fig. 1A and B). At 15 days post agroinfiltration (dpai), the upper non-inoculated leaves of tobacco plants infected with the wild-type PVY showed severe mosaic and distinct veinal necrosis symptoms, while PVY-HC_ELR_ mutant only caused mild mosaic symptoms. The veins of tobacco leaves infected with the wild-type PVY were stained blue by trypan blue dye, while those infected with PVY-HC_ELR_ mutant were not (Fig. 1C). SqRT-PCR results showed that the *CP* RNA accumulation levels of PVY-HC_ELR_ mutant were lower than those of the wild-type PVY in tobacco systemically infected leaves at 15 dpai (Fig. 1D). Western blotting results showed that the CP accumulation levels of PVY-HC_ELR_ mutant amounted to only 53 % of the wild-type PVY (Fig. 1E). These results showed that PVY-HC_ELR_ mutant exhibited attenuated virulence in tobacco plants, and could be a candidate for inducing cross-protection.

### 3.2 PVY-HC_ELR_ mutant could not be transmitted by *M. persicae*

**Fig. 2:**
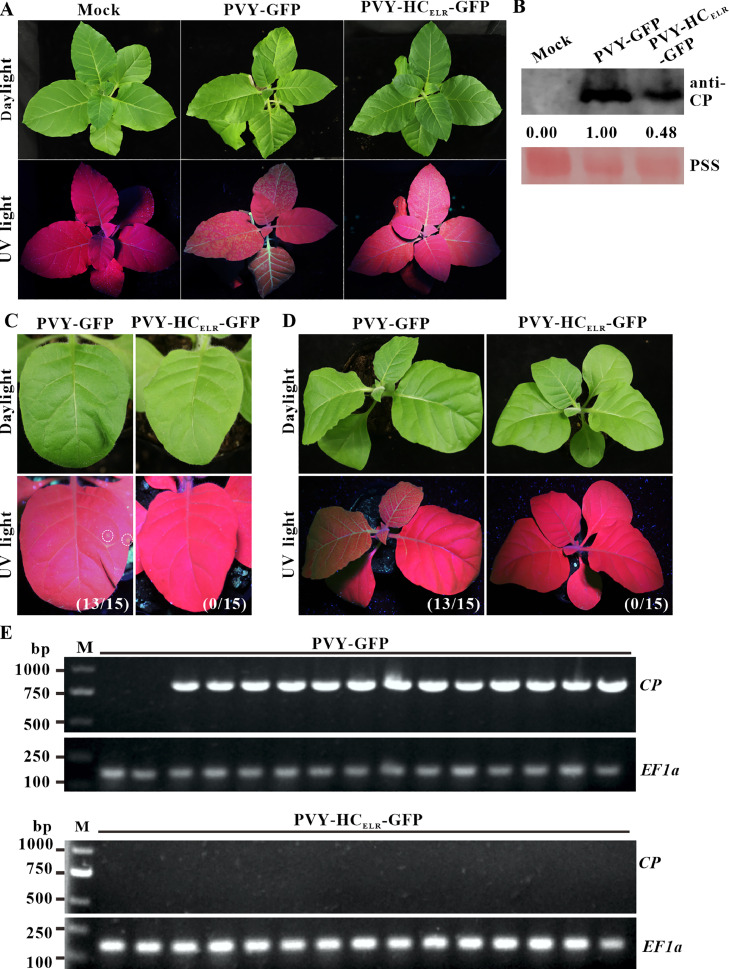
**Aphid transmissibility of attenuated variant of PVY in tobacco plants.** (A) Symptoms under daylight and GFP fluorescent under UV light of tobacco plants inoculated with PVY-GFP or its mutant at 15 dpai. (B) Western blotting analysis of CP accumulation levels of PVY-GFP and its mutant in tobacco leaves at 15 dpai. The sample loadings were shown with PSS. Band intensities were measured using the ImageJ software. Numbers indicated PVY CP accumulation levels normalized to PSS staining. (C and D) Symptoms under daylight and GFP fluorescent under UV light of tobacco plants inoculated with PVY-GFP or its mutant by *Myzus persicae* at 5 d and 10 d. The numbers of symptomatic/inoculated tobacco plants were marked in brackets. (E) SqRT-PCR analysis of *CP* RNA accumulation in tobacco plants inoculated with PVY-GFP or its mutant by *Myzus persicae* at 10 d. Elongation factor 1-alpha (*EF1α*) was used as an internal control.

To investigate whether PVY-HC_ELR_ mutant could be transmitted by *M. persicae*, we constructed plasmid pPVY-HC_ELR_-GFP carrying a *GFP* reporter gene. The tobacco plants inoculated with pPVY-GFP showed apparent mosaic symptoms under daylight and strong GFP fluorescence under UV light, while the tobacco plants inoculated with pPVY-HC_ELR_-GFP only showed mild mosaic symptoms and weak GFP fluorescence at 15 dpai (Fig. 2A). Western blotting results showed that the CP accumulation levels of PVY-HC_ELR_-GFP were substantially lower than those of PVY-GFP in tobacco systemic leaves (Fig. 2B). Aphids were starved for 3 h and then fed with tobacco leaves infected with PVY-GFP or PVY-HC_ELR_-GFP for 5 min. Thereafter, they were transferred onto healthy tobacco plants and allowed to feed on the plants for 12 h. After 5 days of aphid feeding, 13 out of 15 tobacco plants inoculated with PVY-GFP by *M. persicae* showed GFP fluorescent dots, while none of the tobacco plants inoculated with PVY-HC_ELR_-GFP by *M. persicae* showed GFP fluorescent dots (Fig. 2C). After 10 days of aphid feeding, the systemic leaves of 13 out of 15 tobacco plants inoculated with PVY-GFP by *M. persicae* showed mosaic symptoms under daylight and strong GFP fluorescence under UV light, while none of tobacco plants inoculated with PVY-HC_ELR_-GFP by *M. persicae* showed mosaic symptoms and GFP fluorescence (Fig. 2D). SqRT-PCR results showed that PVY-GFP could be transmitted by *M. persicae* on tobacco plants (86.7  % transmission rate), while PVY-HC_ELR_-GFP failed to be transmitted by *M. persicae* (0 % transmission rate) (Fig. 2E).

### 3.3 PVY-HC_ELR_ mutant was stable in tobacco plants

**Fig. 3:**
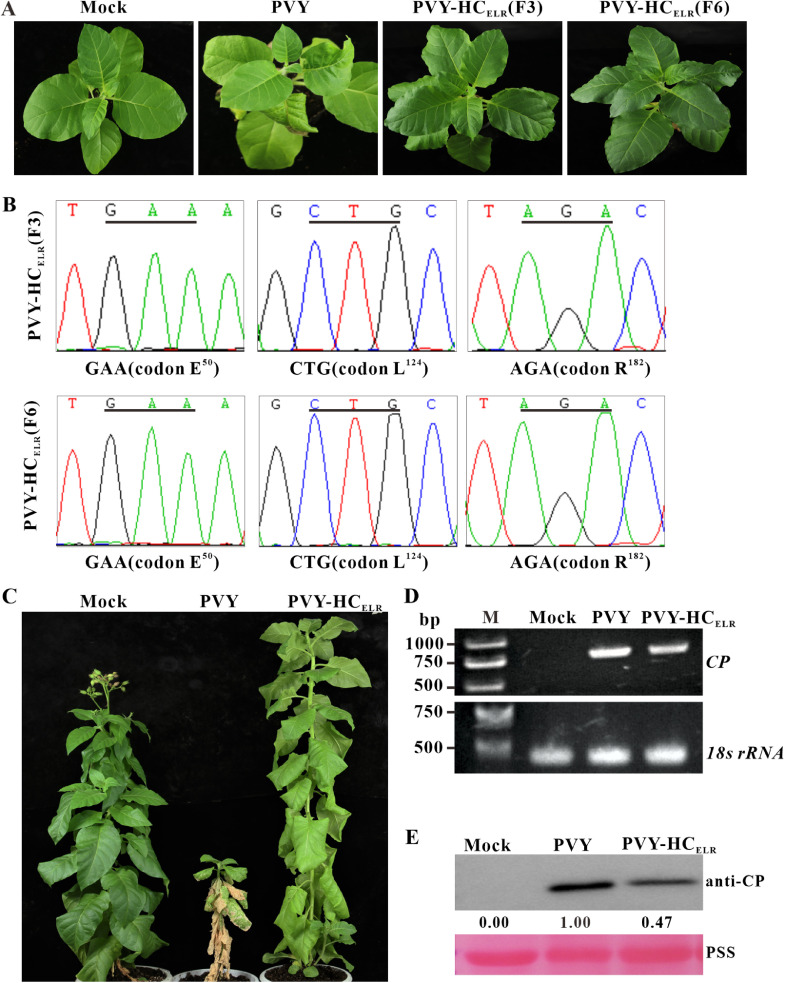
**The stability of PVY-HC_ELR_ mutant in tobacco plants.** (A and B) Symptoms and the sequencing results of HC-Pro RT-PCR products from tobacco plants infected with PVY-HC_ELR_ mutant after three and six serial passages. The sites of point mutations in HC-Pro were underlined and the corresponding amino acid residues were indicated. The images represented at least 12 tobacco plants. (C) Symptoms of tobacco plants infected with the wild-type PVY and PVY-HC_ELR_ mutant at 90 dpai. The images represented at least 12 tobacco plants. (D) SqRT-PCR analysis of the wild-type and mutant PVY *CP* RNA accumulation levels in tobacco upper leaves at 90 dpai. 18S rRNA was used as an internal control. (E) Western blotting analysis of the wild-type and mutant PVY CP accumulation levels in tobacco leaves at 90 dpai. The sample loadings were shown with PSS. Band intensities were measured using the ImageJ software. Numbers indicated PVY CP accumulation levels normalized to PSS staining. The experiments were repeated three times.

To test the stability of PVY-HC_ELR_ mutant, we performed serial passages of progeny viruses from infected plants to healthy tobacco plants at a 20-day interval. The tobacco plants infected with PVY-HC_ELR_ mutant only showed mild mosaic symptoms during six serial passages (Fig. 3A). The HC-Pro encoding sequences of PVY-HC_ELR_ progeny in tobacco systemically infected leaves were sequenced after the three and six serial passages. The sequencing results indicated that PVY-HC_ELR_ mutant was genetically stable in tobacco plants after six serial passages (Fig. 3B).

Tobacco plants inoculated with pPVY and pPVY-HC_ELR_ were cultured until 90 days. The wild-type PVY caused severe dwarfing and leaf necrosis symptoms in tobacco plants, however, tobacco plants infected with PVY-HC_ELR_ mutant grew normally and only showed mild mosaic symptoms at 90 dpai (Fig. 3C). The results of SqRT-PCR showed that compared with the wild-type PVY, the *CP* RNA accumulation levels of PVY-HC_ELR_ mutant decreased in tobacco systemically infected leaves at 90 dpai (Fig. 3D). Western blotting results showed that the CP accumulation levels of PVY-HC_ELR_ mutant were approximately 53 % lower than those of the wild-type PVY (Fig. 3E). All these results suggested that PVY-HC_ELR_ mutant had the potential in cross-protection.

### 3.4 PVY-HC_ELR_ mutant could protect tobacco plants from the severe PVY infection

**Fig. 4:**
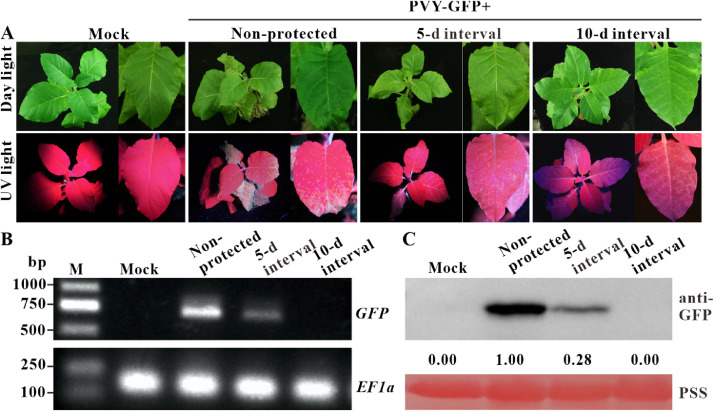
**Attenuated mutant PVY-HC_ELR_ could protect tobacco plants against wild-type PVY infection.** (A) Symptoms of tobacco plants challenged with PVY-GFP at 15 days post-challenge inoculation with intervals of 5 or 10 dpi. Mock, tobacco plants were inoculated with phosphate-buffered saline. Non-protected, tobacco plants inoculated with the empty vector pCB301-Rz. (B) SqRT-PCR analysis of *GFP* RNA accumulation of PVY-GFP in tobacco upper leaves at 15 days post-challenge inoculation. *EF1α* was used as an internal control. (C) Western blotting analysis of the GFP accumulation levels of PVY-GFP in tobacco top leaves at 15 days post-challenge inoculation. Band intensities were measured using the ImageJ software. Numbers indicated PVY CP accumulation levels normalized to PSS staining.

To evaluate the cross-protection efficacy, we pre-inoculated tobacco plants with PVY-HC_ELR_ mutant. At 5- or 10-day protection interval, the first fully expanded leaves of tobacco plants were mechanically inoculated with PVY-GFP. At 15 days post-challenge inoculation, the non-protected tobacco plants showed severe mosaic and distinct leaf necrosis symptoms under daylight and strong GFP fluorescence under UV light. The tobacco plants protected for 5 days by PVY-HC_ELR_ mutant showed apparent mosaic symptoms and GFP fluorescence, while the tobacco plants protected for 10 days by PVY-HC_ELR_ mutant only showed mild mosaic symptoms and no GFP fluorescence in the systemic leaves (Fig. 4A). SqRT-PCR analysis showed he presence of PVY-GFP genome in the control and PVY-HC_ELR_-protected plants with a 5-day protection interval, but not in PVY-HC_ELR_-protected plants with a 10-day protection interval (Fig. 4B). Western blotting results also proved the absence of PVY-GFP in the cross-protected tobacco plants with an interval of 10 days (Fig. 4C). These results indicated that PVY-HC_ELR_ mutant could provide effective protection for tobacco plants against wild-type PVY infection with a 10-day protection interval.

## 4 Discussion

In this study, we developed an attenuated mutant PVY-HC_ELR_ which had three mutations in HC-Pro. The mutant PVY-HC_ELR_ did not cause leaf veinal necrosis and was transmitted by *M. persicae* in tobacco plants. After six successive passages with a 20-day interval, PVY-HC_ELR_ mutant was still genetically stable, and only induced mild mosaic symptoms in tobacco plants even at 90 dpai. This mutant could effectively protect tobacco plants from the wild-type PVY infection. This study provided a promising mild mutant for cross-protection against PVY in tobacco plants.

PVY occurs worldwide and is the major viral threat resulting in high economic losses to crops (Lacomme and Jacquot, 2017; Quenouille et al., 2013). PVY has evolved different strains, including three main strains of PVY^N^, PVY^O^, and PVY^C^, as well as the recombinants PVY^N-Wi^, PVY^NTN^, PVY^N: O^, PVY^NTN-NW^, and PVY^SYR^ (Chikh Ali et al., 2010; Hussain et al., 2016; Jia et al., 2021; Wang et al., 2012; Zhang et al., 2019). PVY^O^ and PVY^C^ usually cause mosaic symptoms in most tobacco cultivars, while PVY^N^ isolates cause severe veinal necrosis symptoms, resulting in yield loss of 39–75 % on tobacco (Tsedaley, 2015). In our study, tobacco plants inoculated with the infectious clone of PVY isolate Guizhou showed severe dwarfing and leaf necrosis symptoms (Figs. 1C and 3C). Cheng et al. (2020) analyzed that PVY isolates Guizhou clustered with isolates of PVY^SYR-I^. Therefore, it is necessary to develop effective mild mutants to protect tobacco plants against PVY^SYR-I^ isolates infection via cross-protection.

Potyviral HC-Pro is pivotal during aphid transmission, RNA silencing suppression, and viral virulence, and also an important target for screening attenuated mutants. Most potyviruses are transmitted by aphids in a non-persistent manner (Nigam et al., 2019; Wylie et al., 2017). The conserved KITC motif in the N-terminus of HC-Pro is critical for virus retention in aphid stylets (Blanc et al., 1998; Huet et al., 1994). Additionally, the conserved PTK motif in the C-terminus of HC-Pro also is essential for the aphid transmission of potyvirus by directly interacting with a highly conserved DAG motif in coat protein (Peng et al., 1998). In this study, by substituting the residue K^50^ in the conserved KITC motif of HC-Pro with E (Fig. 2), we obtained a PVY mutant that is non-aphid transmissible and could avoid the risk of its spreading to non-target crops. Plant RNA silencing is a highly conserved strategy for inhibiting RNA viruses in a sequence-specific manner (Duan et al., 2012; Guo et al., 2016). To ensure successful infection, potyviruses encode HC-Pros to suppress plant antiviral RNA silencing. Previous studies have reported that multiple amino acid residues play important roles in HC-Pro RNA silencing suppression activity and viral virulence (Desbiez et al., 2010; Gal-On, 2000). The amino acid residue asparagine at position 182 (N^182^) in the conserved FRNK motif of HC-Pro was replaced with L reducing its RNA silencing suppression activity and the virulence of soybean mosaic virus (Bao et al., 2020). The mutation of R at position 181 (R^181^) in the FRNK motif of HC-Pro to I reduced the virulence of passiflora mottle virus (Do et al., 2023). In our previous study, we found that the mutations of K^182^ in FRNK motif and K^124^ reduced PVY virulence in tobacco plants (Tuo et al., 2020; Zhang et al., 2021).

Mild strain-mediated cross-protection is a biological strategy to improve plant resistance to virus diseases (Huang et al., 2023). The stability of mild strain is an important limiting factor in the practical application of cross-protection (Costa et al., 2010). Single mutation converted the attenuated strain of pepino mosaic virus into a virulent strain in tomato plants (Hasiów-Jaroszewska et al., 2011). Compensatory molecular evolution of potyviral HC-Pro results in the progenies of mild strains with enhanced virulence (Torres-Barceló et al., 2009). The amino acid residue R in the HC-Pro FRNK motif was substituted with I reduced SCMV virulence. However, a spontaneous complementary mutation of G at position 440 (G^440^) to R in HC-Pro rescued SCMV virulence (Xu et al., 2020). To improve safety, several attenuated mutants with two or more attenuating mutations have been developed (Cheng et al., 2023; Xu et al., 2021). A double-mutant of the passiflora mottle virus was stable after six serial passages in *Passiflora edulis f. flavicarpa* plants (Do et al., 2023). A double mutant of the papaya leaf distortion mosaic virus caused mild symptoms in papaya plants at 60 dpai (Tuo et al., 2020). Two double-mutants of the East Asian passiflora virus were stable after six passages, and provided effective protection against severe strain in yellow passionfruit plants (Chong et al., 2023). In this study, triple-mutant PVY-HC_ELR_ was stable genetically after six passages, and only caused mild symptoms in tobacco plants at 90 dpai (Fig. 3). The PVY-HC_ELR_ mutant could provide complete protection against the wild-type PVY infection in tobacco plants at a 10-day protection interval (Fig. 4).

## 5 Conclusion

Our results demonstrate that the non-aphid-transmissible attenuated PVY-HC_ELR_ mutant provides the material basis for the management of PVY through cross-protection.

## CRediT authorship contribution statement

**Xiao-Jie Xu:** Investigation, Data curation, Writing – original draft. **Xu-Jie Sun:** Investigation, Formal analysis, Data curation. **Chun-Ju Liu:** Methodology, Formal analysis. **Xiu-Zhai Chen:** Validation. **Qing Zhu:** Visualization. **Yan-Ping Tian:** Writing – original draft, Formal analysis. **Xiang-Dong Li:** Writing – review & editing, Project administration, Funding acquisition.

## Declaration of competing interest

The authors declare that they have no known competing financial interests or personal relationships that could have appeared to influence the work reported in this paper.

## Data Availability

Data will be made available on request.

## Appendix Supplementary materials

Image, application 1
