# Supplementary material for: Development of an attenuated potato virus Y mutant carrying multiple mutations in helper-component protease for cross-protection
Source: Virus Res. 2024 Apr 15;344:199369. doi: 10.1016/j.virusres.2024.199369 (PMC11035042; doi:10.1016/j.virusres.2024.199369)
Supplement: Supplementary file 1 [file mmc1.docx]

**Supplemental Table 1: The name and sequence of primers used in this study**

| Purpose | Primer name | Primer sequence (5'-3') |
| --- | --- | --- |
| PVY mutants | PVY-HC-K50E-F | GTGCTATGAAATAACCTGCCCTACCTGCGCCC |
|  | PVY-HC-K50E-R | CAGGTTATTTCATAGCACGGTAAAATACTGTGTGTTATTATCG |
|  | PVY-HC-K124L-F | GGGGAGCTTCAACAATCACCTTTCAAAAACCTGAATATTCTG |
|  | PVY-HC-K124L-R | ATTGTTGAAGCTCCCCTATAGACTTGAATACTTCATTGAAAATTTCTAG |
|  | PVY-HC-K182R-F | TAGGAATCGACTATCTGCCAAAGCAAATTGGAACTTGTATCTG |
|  | PVY-HC-K182R-R | GCAGATAGTCGATTCCTAAAGAACGAGATGTCTCCTTTCT |
| semi-quantitative RT-PCR | PVY-CP-F | GCAAATGACACAATCGATGCAG |
|  | PVY-CP-R | CATGTTCTTAACTCCAAGTAGAG |
|  | *18S rRNA*-F | CGGCGATGCGCTCCTG |
|  | *18S rRNA*-R | TACAGAGCGTAGGCTTGCTTTG |
|  | *EF1α*-F | TGGTGTCCTCAAGCCTGGTAT |
|  | *EF1α*-R | ACGCTTGAGATCCTTAACCGC |
|  | GFP-F | ATGAGTAAAGGAGAAGAAC |
|  | GFP-R | TTTGTAGAGCTCATCCATG |

The underlined letters represented the mutation sites.


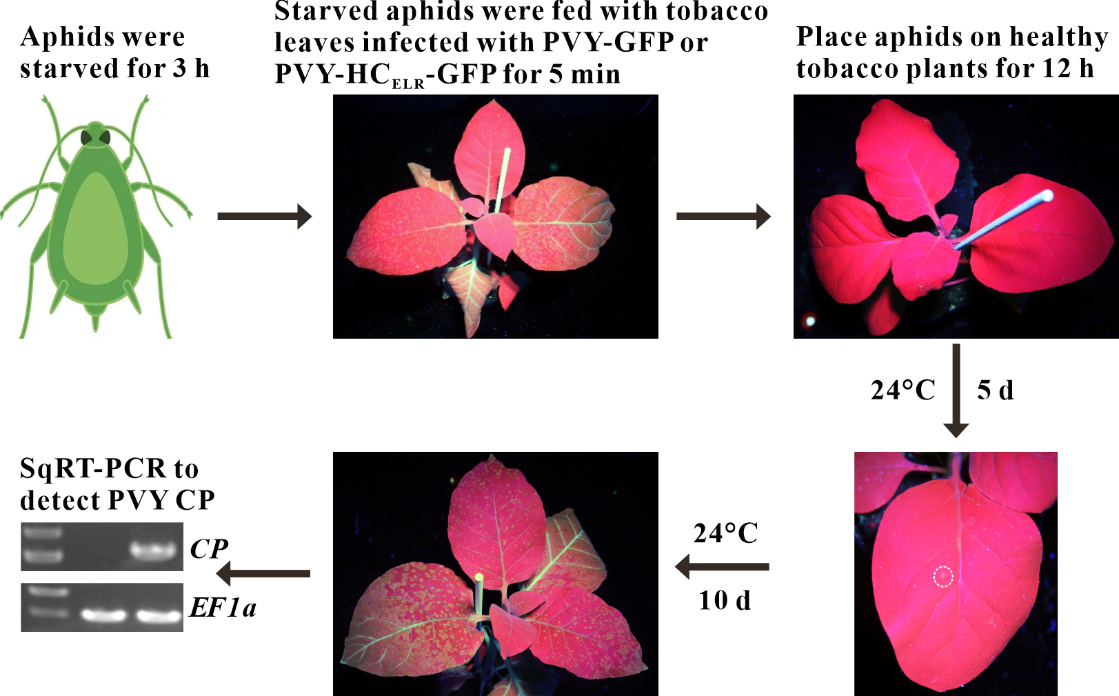


**Supplemental Fig. S1.** The modified method for aphid transmission assays of the wild-type PVY and its mutants in tobacco plants.
